# Enhancing data completeness in time series: Imputation strategies for missing data using significant periodically correlated components

**DOI:** 10.1371/journal.pone.0350666

**Published:** 2026-07-06

**Authors:** Asmaa Ahmad, Eric J. Rose, Michael S. Roy, Edward Valachovic

**Affiliations:** 1 Department of Epidemiology and Biostatistics, College of Integrated Health Sciences, University at Albany, State University of New York, Albany, New York, United States of America; 2 NYS Department of Health, Albany, New York, United States of America; 3 Center for the Elimination of Health Disparities, College of Integrated Health Sciences, University at Albany, State University of New York, Albany, New York, United States of America; Max Planck Institute for Solid State Research, GERMANY

## Abstract

Missing data in periodic time series can bias inference when temporal dependence is not preserved during imputation. We propose a framework that integrates the Variable Bandpass Periodic Block Bootstrap (VBPBB) with multiple imputation using Amelia II by incorporating statistically significant periodic components as auxiliary covariates. Performance was evaluated using simulated missingness in temperature time series data with seasonal structure under a Missing at Random (MAR) mechanism. Imputation accuracy was assessed using Root Mean Square Error (RMSE) and Mean Absolute Error (MAE), comparing Amelia II models with and without VBPBB-derived periodic covariates. Incorporating periodic components reduced RMSE and MAE by approximately 55%, indicating improved reconstruction of seasonal patterns. These results suggest that preserving periodic dependence can enhance imputation performance in time series with strong seasonal structure.

## Background

Missing data present a significant challenge to statistical analyses, complicating inference and reducing accuracy across many applications [1]. In time series data, Missingness can introduce biases that obscure genuine patterns and trends, particularly periodic structures, thereby compromising research validity and predictive performance [2]. This issue is particularly important for datasets with strong seasonal or cyclic behavior, where standard imputation methods may not fully preserve the underlying temporal structure.

To address this issue, we utilize the Variable Bandpass Periodic Block Bootstrap (VBPBB) method [3] to extract statistically significant periodic components from the data. Rather than replacing existing imputation methods, VBPBB is used to capture and preserve underlying temporal structure. This approach builds on the work of Cleveland et al. [4] by emphasizing the preservation of cyclic behavior commonly observed in health-related time series.

This study proposes a structured framework that integrates the Variable Bandpass Periodic Block Bootstrap (VBPBB) with multiple imputation by incorporating statistically significant periodic components as auxiliary covariates. The approach builds on existing methods, including frequency-domain filtering and Amelia II, to better preserve temporal dependence in periodic time series. The resulting improvements are specific to datasets with strong periodic structure.

### Impact of missing data in research

Missing data are a pervasive challenge in research that can compromise the validity of study findings across disciplines. As Bennett [5] highlights, missing data exceeding approximately 10% can introduce bias, distort data structure, reduce statistical power, and lead to inaccurate parameter estimates and standard errors.

To address this issue, several methods have been developed, including multiple imputation (MI), a widely used and robust approach. MI replaces each missing value with multiple plausible values, accounting for uncertainty in the imputation process. Auxiliary variables, those correlated with variables of interest but not included in the primary analysis, can be incorporated to improve imputation accuracy [6,7]. The resulting imputed datasets are analyzed separately and combined to produce overall estimates, thereby reducing bias and improving statistical inference.

When the proportion of missing data is very small, the practical benefits of MI may be less pronounced; however, principled methods such as MI remain preferable when missingness may introduce bias or when imputation uncertainty should be reflected [8]. Historically, researchers often relied on complete case analysis in the presence of substantial missing data [9] although this approach can reduce sample size and statistical power.

More recent work challenges this view. Madley-Dowd et al. [7] demonstrate that MI can remain effective even when a large proportion of data is missing, highlighting the importance of selecting appropriate methods based on the missing data mechanism rather than the proportion alone.

### Types of missing data

Understanding the mechanisms behind missing data is essential for selecting the appropriate methods to address it, as categorized by Rubin [1]:

### Missing Completely at Random (MCAR)

Missing Completely at Random (MCAR) occurs when the probability of missingness is independent of both observed and unobserved variables, meaning that missing values arise purely by chance [10]. Under this assumption, missing data do not introduce bias, making MCAR the least problematic form of missingness. However, verifying the MCAR assumption is important before selecting an appropriate handling method. Statistical tests, such as those proposed by Jamshidian and Jalal [10], assess whether differences in covariance structures across missing data patterns are significant; such differences indicate that the data may not satisfy the MCAR assumption.

Common approaches for handling MCAR data include listwise deletion, pairwise deletion, and mean imputation. Listwise deletion (complete case analysis) retains only observations with complete data and produces unbiased estimates under MCAR, but reduces sample size and statistical power [11]. Pairwise deletion uses all available data for each analysis, preserving more observations, though it may yield inconsistent results if the MCAR assumption is violated [12]. Mean imputation replaces missing values with the variable mean, maintaining a complete dataset but distorting variability and potentially underestimating standard errors [5]. While these methods can effectively address MCAR data without introducing systematic bias, they often reduce the effective sample size, potentially impacting statistical power [5].

### Missing at Random (MAR)

MAR occurs when the probability of missing data is systematically related to other observed variables but remains independent of the missing values themselves. This type of missingness implies a structured pattern that can be predicted based on observed data. As a result, methods such as Multiple Imputation (MI) and Maximum Likelihood (ML) estimation are effective for MAR, as they leverage the relationships within the observed data to impute missing values without introducing significant bias [13,14]. MI, in particular, involves creating multiple plausible datasets and averaging the results to enhance the robustness of the estimates, while ML utilizes all available data to estimate parameters that best fit the observed data structure. The structured nature of MAR allows for reliable prediction of missing values, preserving the relationships between variables within the dataset.

### Missing Not at Random (MNAR)

MNAR is the most complex type of missingness, where the probability of missingness is dependent on unobserved values, making the missing data mechanism inherently related to the missing values. This situation presents a significant challenge, as the missing data is informative and directly related to the unobserved values themselves [1]. Standard imputation techniques are generally inadequate for MNAR, as they fail to account for the bias that the MNAR mechanism introduces. Advanced approaches, such as selection models and pattern-mixture models, are necessary to manage MNAR data. These models explicitly account for the missingness mechanism during analysis, though they require robust assumptions and complex modeling frameworks to address the bias inherent in MNAR data [15].

### Practical assessment and management of missing data

A thorough analysis of the pattern of missingness within a dataset is crucial before implementing any imputation techniques. Such analysis not only helps assess the complexity of the missing data problem but also influences the choice of imputation method, thereby minimizing potential biases.

While the proportion of missing data is an important consideration, it is not the sole factor researchers should assess. According to Tabachnick and Fidell [16], the causes and patterns of missing data significantly impact research outcomes, often more than the proportion of missing data alone. This view is supported by Madley-Dowd et al. [7], who emphasized that the missingness mechanism should guide the selection of an appropriate imputation method.

Their findings suggest that multiple imputation (MI) can produce valid and reliable results even when up to 50% of data is missing, provided the missing data is classified as MAR. This challenges traditional views that high proportions of missing data are inherently problematic and underscores the importance of a detailed approach to data imputation. By understanding both the quantity and the nature of the missing data, researchers can effectively mitigate bias and maintain analytical integrity using appropriate techniques.

### Overview of the imputation process

**Fig 1:**
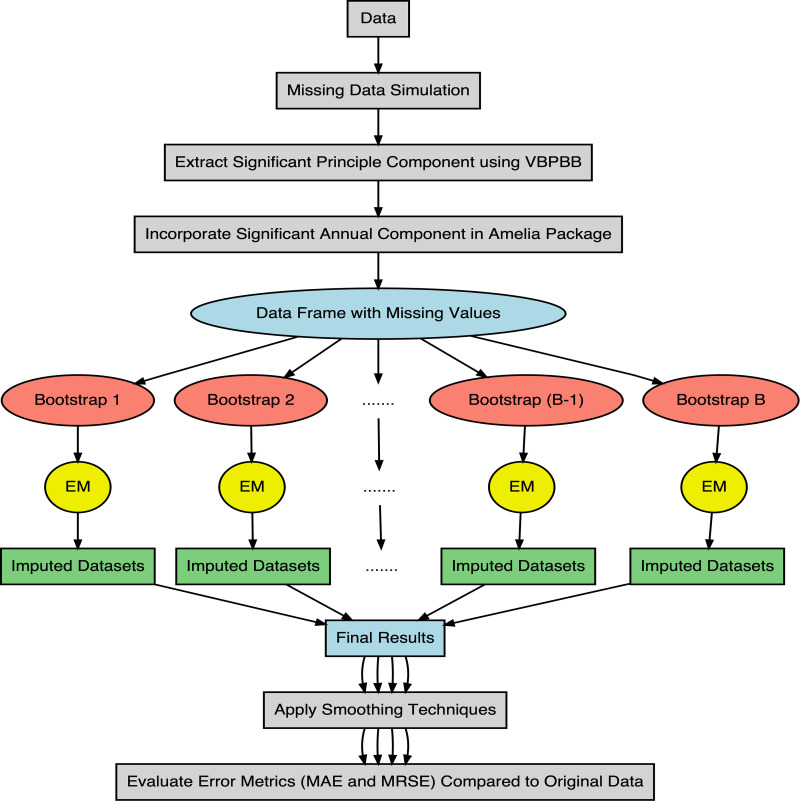
Flowchart of Data Imputation Process Using VBPBB.

To provide a structured framework for understanding the methodology, Fig 1 presents a high-level flowchart outlining the major stages of the data imputation process. The flowchart illustrates the sequential progression from the simulation of missing data to the extraction of significant periodic components using the Variable Bandpass Periodic Block Bootstrap (VBPBB) method, followed by the integration of these components into the Amelia II package for multiple imputation. After imputation, smoothing techniques are applied to refine the reconstructed datasets. The process concludes with an evaluation of imputation performance using Mean Absolute Error (MAE) and Root Mean Squared Error (RMSE) metrics. A detailed description of each step is provided in the subsequent sections.

## Methodology

This study evaluates whether incorporating statistically significant periodic structure improves multiple imputation performance for time series data. Periodic components were extracted using a frequency-domain resampling framework and incorporated as auxiliary covariates in the imputation model. Additional methodological details on the VBPBB framework, KZFT filtering, Amelia II imputation procedure, post-imputation smoothing, and evaluation metrics are provided in S1 File.

### Periodic component extraction using VBPBB and KZFT

Periodic structure was characterized using the Variable Bandpass Periodic Block Bootstrap (VBPBB) framework [3]. In this study, VBPBB was used to extract and reconstruct statistically significant periodic signals that were included as auxiliary covariates in the imputation model. The theoretical development of VBPBB is described in detail in the original source.

Bandpass filtering was implemented using the Kolmogorov–Zurbenko Fourier Transform (KZFT) [17]. For each component, the filter center frequency was set to ν = 1/p, where p is the component period. Filter window width and iteration parameters were selected to retain the target frequency band while attenuating low-frequency trends and high-frequency noise. Components with bootstrap confidence intervals excluding zero amplitude were retained.

To preserve temporal dependence, block resampling was conducted using block lengths equal to the period of each component. The reconstructed periodic vectors summarize dominant cyclical behavior and were incorporated into the Amelia II imputation model as auxiliary covariates.

### Multiple imputation (Amelia II) with and without periodic covariates

Missing values were imputed using Amelia II [18], which implements a bootstrap-based expectation–maximization (EM) multiple imputation procedure under a Missing at Random (MAR) assumption. Amelia II generates m completed datasets by drawing imputations from predictive distributions informed by the observed data and estimated model parameters.

To evaluate the marginal contribution of periodic information, we compared two imputation conditions:

1. **Amelia II + periodic covariates:** Amelia II with VBPBB-derived periodic auxiliary covariates included in the imputation model.
2. **Amelia II baseline:** Amelia II without periodic auxiliary covariates.

### Performance evaluation

Imputation accuracy was quantified using Mean Absolute Error (MAE) and Root Mean Squared Error (RMSE) computed between imputed values and the corresponding known values under the simulation design:


$$\begin{array}{c} MAE=\;\frac{\text{1}}{n}\sum_{i=\text{1}}^{n}\left|y_{i}-{\hat{y}}_{i}\right|,\;RMSE=\sqrt{\frac{\text{1}}{n}\sum_{i=\text{1}}^{n}{\left(y_{i}-{\hat{y}}_{i}\right)}^{\text{2}}} \end{array}$$

Lower MAE and RMSE indicate improved reconstruction accuracy.

### Post-imputation smoothing

LOESS and moving average smoothing were applied after imputation solely to visualize and compare how well each approach preserved long-term seasonal structure. Smoothing was not used to generate imputations and did not affect MAE/RMSE calculations.

### Data simulation for method evaluation

**Fig 2:**
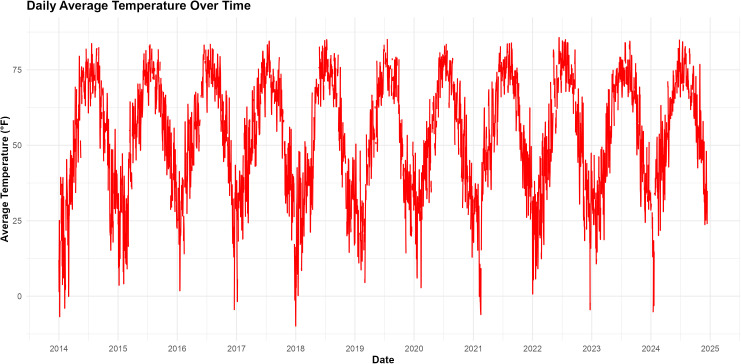
The daily average temperatures from January 2014 to December 2024, as recorded by the Commercial Agriculture Automated Weather Station Network in Albany (Gentry County), Missouri. The temperatures are expressed in degrees Fahrenheit and display clear seasonal fluctuations consistent with regional climatic patterns.

To demonstrate the use of the Amelia II package, incorporating the Variable Bandpass Periodic Block Bootstrap (VBPBB), and to assess the efficacy of data imputation with VBPBB versus imputation methods without VBPBB, we conducted a simulation study within the Missouri Historical Agricultural Weather Database. Specifically, daily average temperature records were extracted from the Commercial Agriculture Automated Weather Station Network, maintained by the University of Missouri Extension [19]. Using a web-based interface, we selected Albany (Gentry County) as the location for our analysis, with the study period specified from January 1, 2014, to December 16, 2024. The complete observed temperature time series used in this analysis is shown in Fig 2.

**Fig 3:**
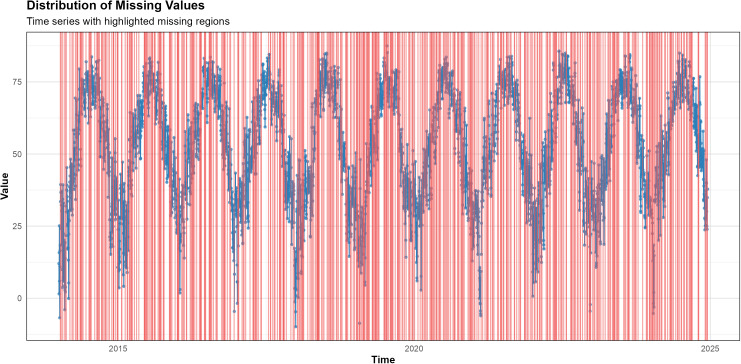
Presents the distribution of missing values in a dataset of daily average temperatures from 2015 to 2025. The graph overlays red vertical bars on a time series plot to indicate days with missing temperature data, while observed temperature values are represented as blue dots.

The simulation was conducted using R version 4.1.1. We categorized days into weekends and weekdays using the wday() function from the lubridate package, assigning 13% of the dataset as missing with a disproportionate number allocated to weekends (30%) to mimic higher non-response rates during these periods. Temperature values for these selected days were designated as missing (NA) through random selection using the sample() function. The resulting distribution of simulated missing values across the temperature time series is presented in Fig 3.

**Fig 4:**
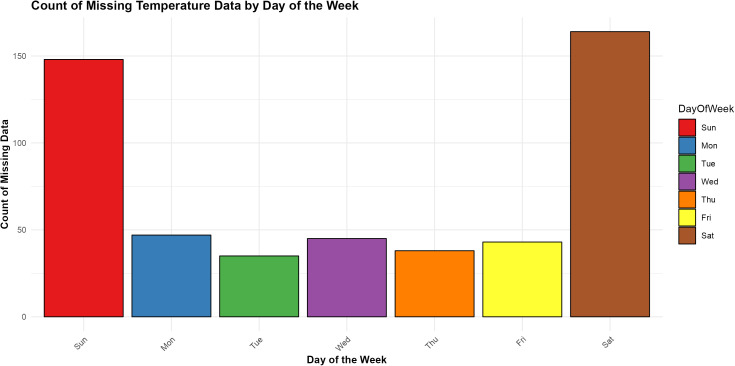
Count of missing data across different days of the week.

Descriptive analysis of missing values is crucial as it helps understand the extent and pattern of missing data within a dataset, ensuring the reliability and validity of subsequent analyses. This involves identifying potential biases and determining the most appropriate strategies for handling missing values. Fig 4 illustrates the proportion of missing data across different days of the week, using distinct colors for each day from Sunday to Saturday. It reveals that a substantial amount of data is missing on weekends, particularly on Sundays and Saturdays, which together account for about 60% of the missing entries. This pattern created to introduce a systematic issue with data reporting or collection during weekends, likely influenced by reduced staffing or operational hours in facilities where the data is gathered. The consistent occurrence of missing data during these times indicates that the data is not missing completely at random (MCAR) but is instead linked to an external factor, such as operational schedules, which does not depend on the unobserved values themselves (i.e., it is missing not at random or MNAR). Given this context, we classify the missing data as Missing at Random (MAR). The missing data is dependent on the day of the week (an observed variable) and not on the unobserved data. This classification allows us to use imputation methods suitable for MAR data to handle the missing values effectively, ensuring robust and reliable statistical analysis. This insight necessitates the use of imputation methods that can account for these systematic missing data patterns to ensure the accuracy and reliability of subsequent analyses.

The extraction of significant periodic components from the temperature dataset using the Kolmogorov-Zurbenko Filter Transform (KZFT) function illustrates an approach for identifying principal components (PCs) associated with both natural and anthropogenic influences. The KZFT function, implemented via the kza package, applies bandpass filters to isolate dominant frequencies corresponding to seasonal and operational cycles, such as daily, weekly, and monthly patterns.

This methodology facilitates the differentiation between naturally occurring cycles, such as seasonal variations driven by atmospheric and climatic processes, and human-induced periodic patterns linked to work schedules, industrial activity, and energy consumption. By selectively filtering and extracting these components, KZFT provides a precise characterization of the underlying drivers of variability within the dataset.

Understanding both environmental and anthropogenic influences enhance the interpretability of time series data, particularly in climate and environmental studies, where distinguishing natural cycles from human-driven impacts is essential for accurate modeling and forecasting. By isolating periodic components at specific frequencies, KZFT enables a comprehensive analysis of temporal patterns, improving the ability to detect, quantify, and interpret seasonal and operational influences in complex datasets.

For the yearly component analysis, a window size of 731 days was used, targeting frequencies like 1/365, 2/365, 3/365, 4/365, 5/365, and 6/365, which capture not only the annual cycles but also biannual and other sub-annual trends. Monthly components were examined with a window size of 101 days at frequencies of 1/30, 2/30, and 3/30, while weekly patterns utilized a window size of 21 days, examining frequencies of 1/7, 2/7, and 3/7. This methodology allows the model to detect a broad spectrum of low-frequency components, extending beyond straightforward cyclic patterns to encompass higher-order harmonics, thereby accommodating more intricate seasonal variations.

To ensure the accuracy and relevance of this analysis, only statistically significant harmonics were retained based on a non-parametric bootstrap procedure. For each extracted periodic component, 95% confidence intervals (CIs) were constructed from the bootstrap samples generated using the Variable Bandpass Periodic Block Bootstrap (VBPBB) method. A periodic component was deemed statistically significant if the maximum lower bound of its confidence interval was greater than the minimum upper bound, indicating that the confidence interval for the periodic component amplitude did not contain zero. This selection criterion ensured that each frequency component contributed meaningful information to the dataset.

After extraction, the isolated principal components were resampled using VBPBB to preserve the integrity of their autocorrelation structures. Notably, the annual and weekly components were identified as statistically significant at the 95% confidence level in characterizing the periodic behavior of the temperature data. The prominence of the annual component aligns with expected seasonal variations driven by solar cycles, reinforcing its critical role in modeling long-term temperature trends. In contrast, the weekly component captures systematic short-term fluctuations likely associated with human or operational factors influencing data collection. Its statistical significance is further corroborated by the observed pattern of increased missing data on weekends, suggesting potential gaps due to reduced staffing, maintenance schedules, or reporting delays. Incorporating this weekly signal into the model enhances the imputation process by preserving short-term temporal dependencies, thereby improving the overall fidelity and robustness of the reconstructed time series.

Following identification of the significant components, median vectors for each were computed to capture the dominant periodic behavior identified through VBPBB. These median vectors were then integrated into the temperature dataset as auxiliary variables to support subsequent imputation using the Amelia II package. In this approach, the significant periodic components provided additional covariate information, allowing Amelia II to align imputations more closely with natural data fluctuations and preserve underlying temporal patterns. This integration was not a separate imputation method but was incorporated directly into the VBPBB-enhanced Amelia II framework. To evaluate the impact of incorporating periodic information, we compared two conditions: (1) Amelia II imputation using auxiliary periodic components derived from VBPBB, and (2) a baseline Amelia II imputation without periodic components. This comparative design allowed for a direct assessment of the added value of periodic information in improving data accuracy.

The effectiveness of each imputation approach was quantified by calculating Root Mean Square Error (RMSE) and Mean Absolute Error (MAE) against actual observed data. The R code used to conduct the simulation, generate the missing data mechanism, extract statistically significant periodic components using KZFT and VBPBB, perform Amelia II imputation with and without periodic auxiliary covariates, and calculate RMSE and MAE is provided in S2 File.

## Results

Our findings, as shown in Table 1, reveal that Amelia Imputation with VBPBB consistently resulted in lower values of Root Mean Square Error (RMSE) and Mean Absolute Error (MAE), with RMSE decreasing significantly from 10.353441to 4.629906, representing a 55.2% reduction, while MAE from 3.014825 to 1.326239, corresponding to a 56% reduction.

**Table 1:** Imputation performance under simulated missingness, reported as mean RMSE and MAE with SD and 95% confidence intervals.

Imputation Method	RMSE	RMSE SD	95% RMSE CI	MAE	MAE SD	95% MAE CI
Amelia Imputation with VBPBB	4.629906	0.60472	[4.516657, 4.685299]	1.326239	0.440219	[1.303253, 1.426019]
Amelia Imputation without VBPBB	10.353441	1.293961	[10.004225, 10.36508]	3.014825	0.957271	[2.885546, 3.152506]

Beyond improved accuracy, the VBPBB-enhanced method demonstrated greater stability, as reflected by the smaller standard deviations (SD) reported in Table 1 across 200 simulation replicates. The reduced variability indicates that performance of the VBPBB method is more consistent under different missing-data realizations. Furthermore, the 95% confidence intervals for both RMSE and MAE did not overlap between methods, confirming that the observed improvements are statistically robust and unlikely to be attributable to simulation variability. These results indicate that incorporating VBPBB-derived periodic components enhances reconstruction reliability by preserving structured periodic behavior that standard imputation alone does not fully capture. The improvements should be interpreted as finite-sample accuracy gains under the specified missingness mechanism rather than as claims of universal or asymptotic superiority.

Additionally, visualization of results was conducted using ggplot2, we generated line graphs to compare the distributions of original and imputed temperature values. This visual assessment was crucial to determine the fidelity of the imputation process and to identify any potential biases introduced.

**Fig 5:**
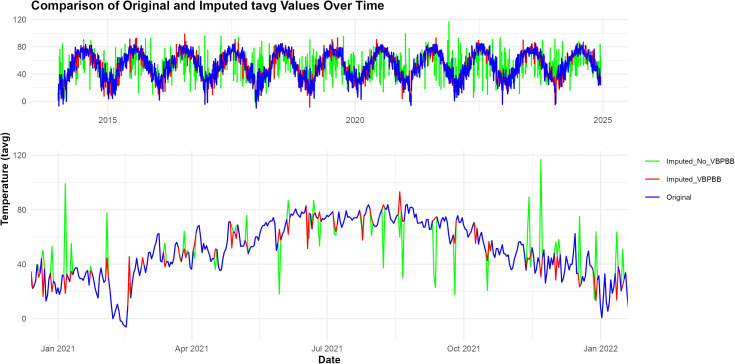
Comparison of Original and Imputed Temperature Values Over Time.

Fig 5 provides a comparative analysis of original and imputed daily average temperature (tavg) values over time, illustrating the effectiveness of different imputation methodologies. The original data (blue line) represents recorded temperature values, while the imputed values using the Variable Bandpass Periodic Block Bootstrap (VBPBB) method (red line) and those imputed without VBPBB (green line) highlight differences in how each approach preserves seasonal patterns and general trends. The top panel spans from 2014 to 2025, providing a long-term view of temperature fluctuations, while the bottom panel focuses on 2021, allowing for a closer inspection of short-term variations.

The VBPBB-enhanced imputation (red line) closely follows the original data (blue line), indicating that this method effectively maintains seasonal cycles and natural variability. In contrast, the imputed values without VBPBB (green line) exhibit greater deviations, particularly in capturing extreme values, suggesting distortions in temperature peaks and troughs. The zoomed-in section further highlights how VBPBB produces smoother and more reliable estimates, whereas the non-VBPBB imputation introduces larger fluctuations and inconsistencies.

The correlation analysis supports these observations, showing a stronger alignment between the VBPBB-imputed data and the original dataset (correlation = 0.9723945) compared to the non-VBPBB imputation (correlation = 0.8618343). This higher correlation confirms that VBPBB more accurately preserves periodic structures and seasonal trends, reducing estimation errors. In contrast, the lower correlation in the non-VBPBB imputation suggests a loss of critical periodic components, further reinforcing the superiority of VBPBB in time series data imputation. These findings demonstrate that VBPBB provides a more robust and reliable approach for handling missing values, ensuring greater accuracy in time-series analysis.

Also, we assessed the quality of the imputed data and the impact of subsequent smoothing techniques, we compare the original temperature data against the imputed and smoothed data using two different smoothing methods.

**Fig 6:**
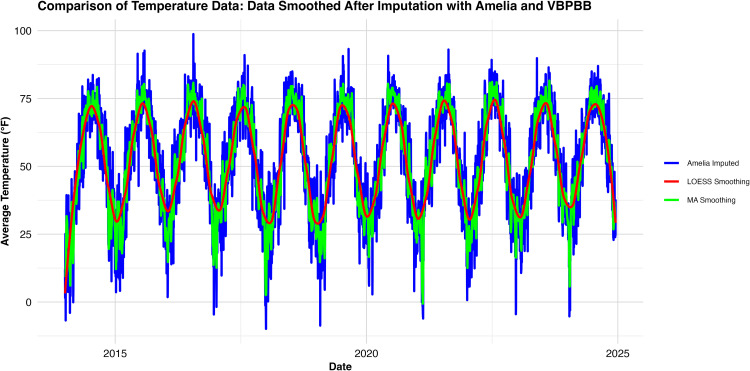
Temperature Data Smoothed After Imputation with Amelia and VBPBB (2014-2025).

Fig 6 shows the original temperature data (blue line) alongside data smoothed using LOESS (red line) and MA (green line) techniques after imputation with VBPBB. The period from 2015 to 2025 highlights how VBPBB affects the imputation’s fidelity to the original temperature trends.

**Fig 7:**
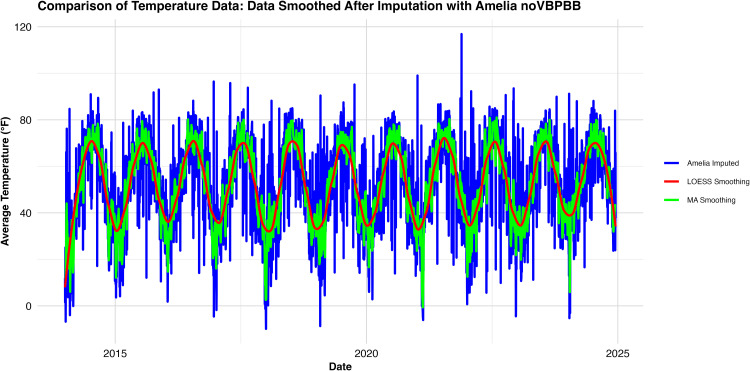
Temperature Data Smoothed After Imputation with Amelia without VBPBB (2014-2025).

Fig 7 presents the original temperature data (blue line) against the LOESS smoothed (red line) and MA smoothed (green line) data after imputation without using VBPBB, illustrating the variance in data smoothing and trend following without the enhancement of VBPBB.

The application of smoothing techniques specifically LOESS (Locally Estimated Scatterplot Smoothing) and Moving Average (MA) serves primarily as a diagnostic and validation tool in this study. These methods are applied after the imputation process to evaluate the effectiveness of different imputation approaches in preserving meaningful temporal structures. Smoothing reduces short-term variability and highlights broader seasonal trends, enabling a clearer visual comparison between the imputed and original temperature series. By comparing smoothed curves from each imputation method both with and without the Variable Bandpass Periodic Block Bootstrap (VBPBB) to the known structure of the original data, we can assess how well the methods maintain long-term and periodic patterns. While smoothing does not improve the imputation itself, it plays a critical role in validating the integrity of the reconstructed time series by helping identify artifacts or distortions introduced during imputation. In this sense, smoothing supports a nuanced evaluation of imputation fidelity, particularly in capturing the expected climatic behaviors embedded in the data.

The results of this analysis reveal that smoothed outputs from VBPBB-imputed data exhibit more consistent alignment with seasonal trends compared to those imputed without VBPBB. Both LOESS and MA techniques help underscore these differences, affirming the utility of VBPBB in maintaining data integrity under conditions of structured missingness. These findings highlight the importance of selecting a robust imputation strategy, particularly for time series with known periodicity, and support the broader applicability of VBPBB in handling the complexities inherent in real-world datasets.

## Discussion

This study introduces a periodicity-aware imputation framework that incorporates statistically significant periodic components into multiple imputation as auxiliary covariates. The framework builds on frequency-domain decomposition, periodic block bootstrap resampling, and multiple imputation. Its main contribution is the integration of these methods to preserve statistically significant periodic dependence during imputation, evaluated through simulation under controlled missingness conditions.

For the periodic time series settings evaluated in this study, augmenting Amelia II with VBPBB-derived components substantially improved finite-sample accuracy. RMSE and MAE were reduced by approximately 56% and 57%, respectively, relative to standard Amelia II. Lower standard deviations and non-overlapping 95% confidence intervals further indicate improved stability across missing-data realizations. These results suggest that incorporating reconstructed periodic components helps preserve seasonal structure, autocorrelation, and cyclic dependence without modifying the underlying Amelia II imputation algorithm.

The framework has several limitations. Its effectiveness depends on the presence of clear periodic structure and may diminish when periodicity is weak. Reliable component identification also requires sufficiently long time series, limiting applicability in short or sparsely observed datasets. In addition, bandpass filtering and block bootstrapping introduce computational overhead and require parameter selection. These challenges are particularly relevant for long, high-frequency series, where repeated filtering and resampling can be computationally intensive, and for short series, where component estimation and block selection may be unstable.

Compared with parametric time-series approaches such as ARIMA models estimated by maximum likelihood, the proposed method does not require specification of a stationary autoregressive structure [20]. Instead, it uses a nonparametric frequency-domain approach to identify and preserve meaningful periodic components, consistent with Kolmogorov–Zurbenko filtering methods [17]. This reduces dependence on fixed model forms and allows greater flexibility in representing complex or irregular periodic patterns. Relative to likelihood-based or machine learning–based imputation approaches, which may require stronger modeling assumptions, large training datasets, or extensive tuning, the proposed framework offers a transparent and interpretable approach for preserving temporal structure [15].

Although this study evaluated the framework under a Missing at Random (MAR) mechanism, periodic time series may also exhibit Missing Not at Random (MNAR) behavior when missingness depends on unobserved values. The proposed method does not explicitly model MNAR mechanisms; therefore, its performance under MNAR conditions remains uncertain. However, incorporating periodic components may help reduce error when missingness is associated with recurring temporal patterns. Future work should evaluate the framework under MNAR mechanisms and across additional empirical datasets.

## Conclusion

This study demonstrates that incorporating statistically significant periodic components into multiple imputation via the Variable Bandpass Periodic Block Bootstrap provides a principled and interpretable framework for improving imputation accuracy in periodic time series. The VBPBB-enhanced approach preserves essential temporal dependence without altering the underlying imputation mechanism, resulting in substantial finite-sample accuracy gains under controlled missingness.

Future work should systematically evaluate the robustness of the VBPBB-enhanced framework across a wider range of missing data proportions and mechanisms, including MCAR, MAR, and MNAR, as well as under varying periodic structures, autocorrelation strengths, and dataset sizes. Extending evaluation to additional application domains, such as environmental and economic time series, will further clarify the generalizability of the approach. While integrating machine learning techniques may improve automation and scalability, the primary strength of the current framework lies in its transparent, frequency-domain treatment of periodic dependence. Continued simulation-based validation will help refine the method and strengthen its role as a reliable tool for addressing missingness in periodic datasets.

## Supporting information

S1 File Supplementary Material for paper 2.docx. This file provides additional methodological details supporting the study, including background on the Variable Bandpass Periodic Block Bootstrap (VBPBB), Kolmogorov-Zurbenko Fourier Transform (KZFT) filtering, Amelia II multiple imputation, post-imputation smoothing techniques, and evaluation metrics used to assess imputation performance.
(DOCX)

S2 File Code for VBPBB_Enhanced Imputation using temperature data.R. This file contains the R code used to prepare the temperature dataset, simulate missing values, extract periodic components, perform Amelia II imputation with and without VBPBB-derived covariates, calculate RMSE and MAE, and generate figures used in the analysis.
(R)
