# Supplementary material for: Enhancing data completeness in time series: Imputation strategies for missing data using significant periodically correlated components
Source: PLoS One. 2026 Jul 6;21(7):e0350666. doi: 10.1371/journal.pone.0350666 (PMC13336469; doi:10.1371/journal.pone.0350666)
Supplement: S1 File — This file provides additional methodological details supporting the study, including background on the Variable Bandpass Periodic Block Bootstrap (VBPBB), Kolmogorov-Zurbenko Fourier Transform (KZFT) filtering, Amelia II multiple imputation, post-imputation smoothing techniques, and evaluation metrics used to assess imputation performance. (DOCX) [file pone.0350666.s001.docx]

**Supplementary Material:**

#### **Variable Bandpass Periodic Block Bootstrap (VBPBB):**

The Variable Bandpass Periodic Block Bootstrap (VBPBB) method, introduced by Valachovic (2024), is designed to analyze time series data characterized by significant periodic components, particularly in cases where traditional methods struggle with inherent instabilities. The VBPBB method and its extension the VMBPBB for multiple periodic components introduced by Valachovic (2025) integrates concepts from Zurbenko’s (1986) iterated moving average bandpass filters to isolate specific frequency components within the data, ensuring a refined and noise-reduced analysis. A key feature of VBPBB is the incorporation of a bandpass filter that targets a specific principal component (PC) frequency, isolating variations at or near the chosen frequency while attenuating those outside the band. This selective filtering enhances the clarity and accuracy of periodic data analysis by reducing the impact of shocks, interventions, noise, outliers, and long-term trends, allowing a sharper focus on essential periodic patterns. By reconstructing a PC time series that retains only the critical correlation structure of a single PC component, VBPBB enables a more precise and structured analysis of periodic data.

Block bootstrapping is a fundamental aspect of VBPBB, ensuring that the correlation structure of the time series is preserved while resampling. By selecting a block size that matches the period of the PC component, VBPBB maintains temporal integrity and improves estimation precision. This results in smaller confidence interval (CI) sizes for the periodic mean compared to traditional periodic block bootstrap methods, as supported by Brockwell and Davis (2002), who emphasize the importance of maintaining dependency structures in time series resampling. Furthermore, spectral analysis, particularly through periodograms, plays a crucial role in identifying dominant periodic components, enhancing the method’s effectiveness in extracting relevant patterns. The collaborative work of Valachovic and Shishova (2024) highlights how periodograms aid in uncovering key periodic structures within complex datasets.

In practical applications, VBPBB isolates major periodic components such as annual or seasonal variations, preserving original correlation structures while ensuring a clearer interpretation of cyclic patterns. This selective filtering and resampling process significantly improves upon traditional resampling techniques, providing a more precise, data-driven, and methodologically rigorous approach to periodic time series analysis. By focusing exclusively on relevant frequency components, reducing noise interference, and preserving temporal dependencies, the VBPBB method offers a robust and flexible solution for capturing intricate temporal dynamics, making it an invaluable tool for researchers working with periodic time series data. The choice of the VBPBB approach was informed by its unique capability to preserve the temporal autocorrelation structures inherent in periodic data, overcoming limitations observed in traditional approaches, which typically neglect these critical correlations.

### KZFT Filters Application

As part of the VBPBB implementation, the Kolmogorov-Zurbenko Fourier Transform (KZFT) is applied to principal component (PC) time series data to achieve the selective filtering necessary for isolating key spectral components. The KZFT method, which integrates elements of moving averages and convolution within the time domain (Zurbenko, 1986), decomposes a time series into its constituent frequency components by applying a series of weighted moving averages. This iterative smoothing process extracts frequency content information at multiple scales, allowing for precise analysis of periodic structures.

In VBPBB, the frequency center (ν) for each KZFT filter is set to the reciprocal of the period (*1/p*), where p denotes the period of the PC component of interest. Additional parameters, such as the width of the moving average filter window (*m*) and the number of filter iterations (*k*), are carefully selected to ensure the filter passes only the desired PC component frequency (Yang and Zurbenko, 2007). When analyzing two or more PC components, strategically placed cutoff frequencies between PC component frequencies are used to enhance separation (Valachovic, 2025). This approach refines the dissection and analysis of periodicity, contributing significantly to the methodological advancements achieved through the VBPBB framework.

**Amelia II**:

Amelia II, developed by Honaker, King, and Blackwell (2011), is an R package designed for multiple imputation. This statistical technique addresses missing data within complex datasets, including those with time series and cross-sectional elements. Amelia II utilizes a bootstrapped version of the Expectation-Maximization (EM) algorithm, integrated with advanced statistical modeling, to effectively manage missing data characterized by non-response and attrition. It operates under the assumption, common to most multiple imputation methods, that data are missing at random (MAR). This means the pattern of missingness depends only on the observed data, *Dobs*, and not on the unobserved data, *Dmis.*

The EM algorithm in Amelia II involves:

- **Expectation Step (E-step)**: Calculates the probabilities of missing data points based on the observed data and current estimates of model parameters.
- **Maximization Step (M-step)**: Updates the parameters to maximize the likelihood of the data, integrating the imputed values into the dataset seamlessly.

Zhang (2016) describes the Bootstrap-based EM algorithm as a sophisticated method for handling missing data, ideal for generating multiple imputations. The process begins by creating multiple samples from the original dataset; specifically, it draws *m* samples, each with n observations, where *n* is the size of the original dataset. For each sample, the EM method calculates point estimates of the mean and variance. Using these estimates, missing values are then imputed by drawing from predictive distributions based on the estimated parameters, ensuring that each imputation appropriately reflects the uncertainty in the missing data (Honaker et al., 2011). These imputed values are inserted into the original dataset, resulting in *m* complete datasets ready for further analysis.

Amelia II enhances imputation accuracy by utilizing bootstrapping, generating multiple samples of the original dataset through resampling with replacement and applying the EM algorithm to each. This not only improves the accuracy of imputations but also provides crucial estimates of variability, essential for robust statistical analysis.

Particularly skilled at handling time series data, Amelia II ensures that imputed values maintain essential temporal correlations, seasonal patterns, and trends. It incorporates ARIMA models within the EM algorithm for a nuanced treatment of datasets with inter-temporal dependencies. Moreover, the integration of significant periodic components through methods like the Variable Bandwidth Periodic Block Bootstrap (VBPBB) allows for adjustments for temporal and nonlinear patterns, enhancing the quality of imputation.

The flexibility of Amelia II enables efficient handling of large and complex datasets, accommodating variables not affected by missingness to refine the imputation process further. Coupled with the provision of uncertainty measures around point estimates, Amelia II stands out as a valuable tool for researchers dealing with incomplete data in time series analysis. By leveraging VBPBB for precise periodic identification, Amelia II enhances the imputation process by treating significant periodic components as auxiliary variables. This adjustment for temporal and nonlinear patterns significantly reduces the risk of bias introduced by inappropriate handling of time-related dependencies.

Amelia II was intentionally selected as the baseline imputation method because it is well established to perform robustly for time series data; the comparison with and without VBPBB was designed to isolate the marginal contribution of incorporating statistically significant periodic components, rather than to benchmark across fundamentally different imputation algorithms.

Formally, missing values are imputed from the conditional distribution

$$X_{mis}|X_{obs}, \hat{P}\left( t \right),$$

where $\hat{P}\left( t \right)$denotes statistically significant periodic components reconstructed via the Variable Bandpass Periodic Block Bootstrap (VBPBB) and incorporated as auxiliary covariates within Amelia II’s bootstrap-based EM algorithm. In this formulation, VBPBB does not modify the imputation mechanism itself, but supplies structured temporal information that constrains the conditional model used for imputation.

### Application of Smoothing Techniques

To refine our analysis, various smoothing techniques, including LOESS smoothing, and moving averages were applied uniformly across the entire dataset after initial preprocessing, which adjusted for seasonality and trends. Each technique was chosen for its ability to reduce noise while preserving essential data characteristics.

**Moving Average:** This technique smooths out short-term fluctuations and highlights longer-term trends or cycles in time series data. By calculating the average of data points within a specified moving window, it effectively filters out 'noise' and clarifies the underlying trend (Box et al., 2015). This window can be centered, depending on the arrangement of the data points relative to the time of prediction (Isnanto, 2022). Moving averages are particularly useful for datasets with seasonality, as adjusting the window size to match the length of the seasonal pattern can help mitigate seasonal variations.

**LOESS (Locally Estimated Scatterplot Smoothing):** is a non-parametric method that uses local polynomial regression to smooth fluctuations in data. Isnanto (2022) discussed that LOESS can fit more flexible shapes because it uses a localized fitting technique, where each data point is smoothed by considering only the nearby points defined by a smoothing parameter. This parameter controls how much the surrounding points influence the smoothing, allowing LOESS to adapt to changes in the data's statistical properties over time.

In time series analysis, two popular smoothing techniques are filtering, often involving averaging, and local regression, as discussed by R. Rizal Isnanto (2011). Both methods utilize a concept called a span or window size, which specifies the range of neighboring data points considered for smoothing at each data point across the dataset. As this window of points moves through the dataset, it calculates the smoothed value for each point. Isnanto points out that the size of the span significantly impacts the results: a larger span results in smoother data but with reduced detail (resolution), whereas a smaller span provides finer details at the cost of smoothness. He emphasizes that determining the ideal span size generally involves trial and error, as it varies based on the characteristics of the dataset and the specific requirements of the smoothing method employed.

**Evaluation Metrics for Imputation Methods: MAE and RMSE**

For assessing the accuracy and reliability of our imputation methods, we also employed Mean Absolute Error (MAE) and Root Mean Squared Error (RMSE). These metrics measure the deviation of imputed values from actual observations, offering a quantitative basis for comparing the performance of different imputation techniques:

1. **Mean Absolute Error (MAE):** calculates the average of the absolute differences between the predicted (or imputed) and actual values across all observations. A lower MAE indicates that the predictions are closer to the actual outcomes, demonstrating better performance of the imputation method.

It is defined as:

$$MAE= \frac{1}{n}\sum_{i=1}^{n} \left| y_{i}-\hat{y}_{i} \right|$$

where $y_{i}$ represents the actual values, $\hat{y}_{i}$ denotes the predicted or imputed values, and *n* is the number of data points. A lower MAE indicates better performance, showing that predictions are closer to actual outcomes (Willmott & Matsuura, 2005).

1. **Root Mean Squared Error (RMSE):** provides a measure of the average magnitude of the errors, penalizing larger errors more heavily due to the squaring of the error terms. It is particularly useful when large errors are undesirable in the analysis.

The formula for RMSE is:

$$RMSE=\sqrt{\frac{1}{n}\sum_{i=1}^{n} {(y_{i}-\hat{y}_{i})}^{2}}$$

A lower RMSE indicates a closer fit of the model to the data, suggesting better performance of the imputation method (Chai & Draxler, 2014).

These metrics are critical not only for evaluating the immediate accuracy of the imputed data but also for ensuring that the methods used adhere to the expected standards of reliability and precision in statistical analysis. Because imputation performance is evaluated against known held-out values generated under a controlled missingness mechanism, cross-validation is not directly applicable in this setting.
